# Fracture prediction using 3D-DXA-finite element based femoral strength: a prospective study in postmenopausal women

**DOI:** 10.1016/j.bonr.2026.101906

**Published:** 2026-02-10

**Authors:** Yvan Gugler, Philippe Zysset, Serge Ferrari, Emmanuel Biver

**Affiliations:** a ARTORG Center for Biomedical Engineering Research, University of Bern, Switzerland; b Division of Bone Diseases, Geneva University Hospitals and Faculty of Medicine, University of Geneva, Geneva, Switzerland

**Keywords:** Femoral strength, DXA, 3D DXA, Finite element analysis, Osteoporosis, Fracture prediction

## Abstract

Although areal bone mineral density (aBMD) measured by DXA is a good predictor of fractures, nearly half of low-trauma fractures occur in individuals without osteoporosis (T-score > −2.5 SD). This study investigated whether femoral strength estimated from 3D DXA-based finite element (FE) analysis enhances the prediction of incident fractures compared with conventional 2D DXA-derived aBMD. Baseline hip DXA scans from 740 postmenopausal women in the Geneva Retirees Cohort were analyzed. 3D reconstructions of the proximal femur were generated to estimate femoral strength using FE analysis and to derive structural bone parameters. Over a mean follow-up of 5.7 ± 1.5 years, 100 low-trauma fractures were recorded, including 44 major osteoporotic fractures (MOF), 89% at non-hip sites. Femoral strength, total and trabecular vBMD, some cortical parameters, and conventional areal BMD were all significantly associated with incident low-trauma fractures (risk increase of 23–38% per SD decrease) and major osteoporotic fractures (36–59%), with femoral strength showing hazard ratios (95% confidence interval) of 1.32 (1.08–1.60) and 1.50 (1.12–2.01), respectively. A strength threshold of 2600 N improved fracture risk classification beyond conventional aBMD thresholds and outperformed the previously proposed fragile strength 3000 N threshold. Notably, 25% of women with low-trauma fractures and 27% with MOF were non-osteoporotic by aBMD but exhibited fragile femoral strength (≤3000 N), with 14% and 16% respectively below the very fragile threshold (≤2600 N). In conclusion, 3D DXA-derived femoral strength provides complementary value to aBMD in identifying women at elevated fracture risk, particularly those not classified as osteoporotic by conventional criteria.

## Highlights

• Femoral strength estimated from 3D DXA predict incident low-trauma fractures and major osteoporotic fractures.
• A strength threshold of 2600 N improved fracture risk classification beyond the usual 2Dbone density threshold (hip T-score ≤ –2.5 SD).
• 25% with low-trauma fractures and 27% with MOF were non-osteoporotic but had fragile femoral strength (≤3000 N).

## 1 Introduction

Osteoporosis is currently diagnosed through the measurements of areal bone mineral density (aBMD) by dual energy X-ray absorptiometry (DXA). Yet DXA is limited for fracture prediction as it does not completely capture all bone strength components, since reduced bone strength reflects the integration of bone mass but also bone structure and geometry. aBMD summarizes all densitometric information in a single scalar measure from a 2D projection of the three-dimensional geometry (Dall'Ara et al., 2013a). Consequently, it does not capture shape nor the distribution of bone mineral that contribute to strength (Keaveny et al., 2020). Finite element analysis (FEA) based on quantitative computed tomography (QCT) produces more accurate estimates of bone strength through the generation of a 3D structural model, which accounts for geometry and mineral distribution (Keyak et al., 1998; Dall'Ara et al., 2013b; Orwoll et al., 2009). QCT-based FEA of the hip demonstrated similar ability to predict major osteoporotic fractures (MOF) as DXA (Amin et al., 2011; Michalski et al., 2021; Bouxsein et al., 2020; Fleps et al., 2022). In addition, prior cohort studies, including the cohort used in the present analysis, demonstrated that peripheral bone strength assessed at the distal radius or tibia using FEA based on high resolution peripheral QCT, is slightly more predictive of fractures than hip or femoral neck aBMD from DXA (Biver et al., 2018).

However, implementing QCT and its derived FEA as a standard tool for bone strength assessment is not feasible, given its limited availability, higher cost, and substantially greater radiation exposure compared with DXA (Keaveny et al., 2020). 3D DXA images, which exploit statistical shape and appearance models (SSAM) to produce QCT-like images based on standard DXA images, offer an alternative input for this kind of analysis (Humbert et al., 2017; Ruiz Wills et al., 2019). Ex-vivo comparisons of femoral strength showed a good correlation between results obtained with 3D DXA and QCT-based FEA (Dudle et al., 2023). High correlations (R^2^ 0.86) have also been reported between the femur strength predicted by QCT-FE and 3D-DXA-FE models in a clinical cohort of subjects aged between 23 and 91 years (Qasim et al., 2024). Moreover, some 3D DXA implementations generate a comprehensive summary of proximal femur densitometric and structural parameters of the proximal femur (Humbert et al., 2017; Humbert et al., 2016; Clotet et al., 2018). Such structural properties are known from DXA or CT-based hip structural analysis (HSA) and have been associated with hip fractures independently from aBMD (Beck et al., 2000; Kaptoge et al., 2008; Ito et al., 2011; Johannesdottir et al., 2013; Borggrefe et al., 2016). 3D-DXA-derived trabecular (Tb) and integral volumetric BMD (vBMD) parameters at the proximal femur showed a better ability than femoral neck (FN) aBMD but not total hip (TH) aBMD for the prediction of hip fractures (Iki et al., 2021). A recent FEA that used SSAM-based 3D reconstructions of hip DXA scans with another method as the present study, outperformed DXA in hip fracture prediction (Grassi et al., 2023; Grassi et al., 2025).

Thus, since 3D-DXA-based finite element analysis (FEA) might provide complementary biomechanical determinants of bone fragility that are not captured by conventional 2D-DXA alone, we aimed to test its ability to provide incremental value beyond standard DXA measurements for predicting major osteoporotic fractures (MOF). The performance of 3D DXA images and thereon FEA-based hip strength assessment for the discrimination of subjects regarding future non-hip fracture occurrence, which are more prevalent than hip fractures in younger populations, has not been investigated. It also remains unclear whether a threshold of hip mechanical fragility can identify fracture risk in individuals with non-osteoporotic bone mineral density (T-score > −2.5 SD).

We applied 3D-Shaper, a 3D DXA algorithm, and a previously developed FEA pipeline to reconstruct 3D DXA images and compute hip strength from hip DXA scans in postmenopausal women from the Geneva Retirees Cohort (GERICO) (Dudle et al., 2023). We explored how 3D DXA-based FEA and structural parameters of the hip predict incident MOFs and low-trauma (LT) fractures and compared them to standard DXA measures.

## 2 Material and methods

### 2.1 GERICO cohort

GERICO (http://www.isrctn.com/ISRCTN11865958), a prospective cohort study running from February 2008 to December 2018 aimed at identifying determinants of musculoskeletal aging. Through multiple recruitment pathways, i.e., mass mailings, advertisements in local newspapers, among hospital staff and in large companies, 833 healthy community-dwelling postmenopausal women, aged 63 to 67 years, were recruited between July 2008 and December 2014 for a baseline visit (Biver et al., 2018; Hars et al., 2016). Patients who suffered from comorbidities known for affecting bone health were not included in the study. Exclusion criteria encompassed a history of cancer with treatment in the past 5 years, renal failure, liver or lung disease, hyperparathyroidism, Paget disease, malabsorption, corticosteroid therapy and any neurological and musculoskeletal conditions possibly affecting bone health (Hars et al., 2016). Ethics approval for the study protocol was granted by the Geneva University Hospital's Ethics Committee. All subjects needed to provide written informed consent.

### 2.2 BMD assessment

TH and FN aBMD were assessed at baseline in all participants using standard DXA on a device of type Hologic QDR Discovery (Hologic Inc., Waltham, MA, USA).

### 2.3 3D DXA models

3D DXA reconstructions of all available DXA images were generated using the 3D-Shaper software (version 2.9.0, 3D-Shaper Medical, Barcelona, Spain). The 3D-Shaper output encompasses a patient-specific 3D image with voxel values corresponding to the local volumetric BMD (vBMD) and a surface mesh of the periosteal surface (Humbert et al., 2016) 3D-Shaper evaluates cortical thickness and density at each vertex in the surface mesh. Bone mineral content (BMC), vBMD and volume are computed in trabecular (Tb) and cortical (Ct) compartments and their union (integral). This is done in the regions of the proximal femur corresponding to the standard regions of interest in conventional DXA scanning: FN, trochanter, shaft and TH corresponding to the union of the three previous ones (Henney et al., 2024). Average cortical thickness (CTh) and surface density (sBMD), the product of cortical vBMD and CTh, were computed for FN, intertrochanteric (IT) and shaft sections and for the union of the three (TH). In addition, these variables were evaluated for medial (med) and lateral (lat) parts of the three regions separately (Humbert et al., 2017; Shen et al., 2024). Finally, the slice area (SA), cross-sectional area (CSA), the cross-sectional moment of inertia (CSMI) and the section modulus Z were evaluated at FN and IT sections (Clotet et al., 2018). In short, SA is the area of the cross-section, CSA or bone area is the density-weighted area of the cross-section, CSMI is the density-weighted polar moment of inertia and Z is obtained by dividing CSMI by the maximum distance from the cross-section center of mass to the circumference. CSA reflects the capacity of the section to resist compression, whereas CSMI and Z reflect the capacity to resist bending (Khoo et al., 2005). An overview of the included parameters with their abbreviations is given in the Supplementary Table 1. The different regions for the evaluation of densitometric and structural parameters are shown in Supplementary Fig. 1A.

### 2.4 FE analysis

The FE methodology has been described elsewhere and is briefly summarized (Dudle et al., 2023). Following the definition of an internal coordinate system of the proximal femur consisting in two axes (neck and proximal shaft), the bone mask was transformed to a position mimicking an impact on the greater trochanter (GT). The shaft axis was inclined by 10° with respect to the horizontal. The plane formed by neck and shaft axes was oriented vertically. Following downsampling to an isotropic voxel size of 3 mm, each element was assigned elastic and yield properties based on the local μCT-equivalent bone volume fraction (BV/TV) computed from vBMD values in the same region (Dall'Ara et al., 2013b). Voxels were directly converted to linear hexahedral elements (C3D8) with elastic-perfectly plastic material behavior including isotropic damage (Schwiedrzik and Zysset, 2013). Nodes at the distal end of the proximal femur were completely constrained. Padding elements mimicking a polyurethane embedding in a steel cup were added at the femoral head and GT. The padding was introduced to mimic a previous experiment and fulfilled the function of redistributing the load on a larger surface (Dall'Ara et al., 2013b). A displacement boundary condition was applied to a reference node in the center of the femoral head, which was coupled to the padding layer on top of the femoral head (Panyasantisuk et al., 2018). FE simulations in fall and stance load configurations, were performed using the commercial FE solver Abaqus 2021.HF4 (Dassault Systèmes, Vélizy-Villacoublay, France). Strength was defined as the reaction force when a displacement corresponding to 4% of the distance femoral head center – GT was reached (Orwoll et al., 2009). Similarly, a load case corresponding to the stance phase of physiologic gait was defined. An illustration of both load cases can be found in Supplementary Fig. 1 B—C.

### 2.5 Fracture assessment

Fractures were assessed through structured face-to-face interviews during follow-up visits, telephone interviews, by mail or medical reports. We requested written confirmation (e.g., reports from radiologists) for all incident fractures. Fractures were reassessed and confirmed by a medical doctor of the study. Primary and secondary outcomes were incident clinical LT fractures and MOFs, which included humerus, forearm, proximal femur, and vertebral fractures. The definition of LT fractures encompassed all fractures resulting from a fall from standing height or less, with the exclusion of finger, toe, skull, and face fractures. Women who experienced traumatic fractures (except fingers, toes, skull, and face) or had vertebral deformity without clinical symptoms nor radiographic confirmation were excluded from the fracture analysis. The control group for the subsequent analyses was composed of the women without incident fractures (except finger, toe, skull, and face fractures).

### 2.6 Anatomical distribution of cortical parameters

We used the surface meshes with individual distributions of CTh and sBMD generated for each subject to test for anatomical differences between controls and subjects who sustained MOFs. Local Ct vBMD was computed by division of local sBMD and CTh values at each node in the FN, trochanter and shaft regions. Average CTh, Ct sBMD and Ct vBMD at each node were computed for the subjects with MOFs and for the control group.

### 2.7 Statistical analysis

Statistical analysis was performed using the software STATA, version 14.0. (StataCorp LP,

College Station, TX, USA) and Python 3.10.9 using the libraries SciPy, NumPy, statsmodels and Pandas.

We computed means and standard deviations of densitometric parameters at baseline for controls without incident fractures, women with LT clinical fracture and women with MOFs. Group differences were assessed using a Mann-Whitney test.

Using Cox proportional hazard's model, we computed hazard ratios (HR) for time to first MOF or LT clinical fracture per standard deviation (SD) decrease in the DXA, 3D DXA and FEA-based parameters. Age-adjusted Harrell's C index was computed as a measure of the predictive power of the two best 3D DXA parameters among FN and TH regions and 3D DXA-based FE strength and compared to DXA-based FN and TH aBMD (Harrell Jr. et al., 1982).

Kopperdahl et al. introduced a threshold of 3000 N for fragile femoral strength in women, which mimics the DXA-based T-Score threshold of −2.5 for osteoporosis (Kopperdahl et al., 2014). We reused the threshold to perform a reclassification analysis. Net reclassification improvements (NRI) for MOFs and LT clinical fractures were computed comparing the discrimination ability between two logistic regression prediction models, adjusted for follow-up duration, using FN aBMD- respectively TH aBMD-based T-score ≤ −2.5SD ± strength classes (Pencina et al., 2008). To account for the underestimation of strength by 3D DXA-based FEA when compared to QCT-based FEA found by a recent study, NRIs were also computed for alternative thresholds ranging from 2300 to 2900 N (Dudle et al., 2023). For calculation of NRI, the cutoff probabilities separating fracture risk categories were fixed at 15% for MOF and 20% for LT clinical fractures, based on a priori and clinically meaningful risk categories intervention cutoff according to those recommended by the Swiss Association against Osteoporosis at the age of 65 (18%) (Mühlenbruch et al., 2013).

Univariate and multivariate Cox-regressions analyses using various thresholds of strength and aBMD were performed and Harrell's C statistics were used to compare fracture discrimination between models.

## 3 Results

Eight hundred and thirty-three post-menopausal women were initially recruited; 93 were excluded for missing DXA data (*n* = 9), loss of follow-up (*n* = 34), or because of incident traumatic fracture (*n* = 50). The present analysis was conducted on 740 women prospectively evaluated over 5.7 ± 1.5 years after the baseline examination for the occurrence of LT clinical fractures (Supplementary Fig. 2). One hundred women experienced incident clinical LT fractures, including 44 women with a MOF (forearm *n* = 19, humerus *n* = 16, proximal femur *n* = 5, vertebra *n* = 4) (Supplementary Table 2). Women with incident LT clinical fractures and MOFs had consistently lower values for aBMD (−4 to −5% and − 6%, respectively), strength from 3D DXA-based FEA (−8 to −9% and − 11 to −14%, respectively) and 3D DXA-based BMD and structural parameters (−2 to −7% and − 2 to −13%, respectively) (Table 1). Only 15% of the women experiencing a LT clinical fracture or MOF had osteoporosis as assessed by FN or TH T-scores.

**Table 1:** Characteristics of women at baseline with and without incident fractures.

	No incident fractures (*n* = 640)	Incident low-trauma clinical fracture	*P*-Value	Incident major osteoporotic fracture	P-Value
**Women characteristics**					
Age [years]	65.1 ± 1.5	65.1 ± 1.4	0.963	64.9 ± 1.4	0.611
Body mass index [kg/m^2^]	25.1 ± 4.5	25.4 ± 4.8	0.556	25.2 ± 4.7	0.802
**DXA areal BMD**					
TH aBMD [g/cm^2^]	0.849 ± 0.112	0.813 ± 0.112	**0.006**	0.800 ± 0.110	**0.013**
TH T-Score [SD]	−0.76 ± 0.92	−1.06 ± 0.92	**0.006**	−1.16 ± 0.91	**0.013**
FN aBMD [g/cm^2^]	0.713 ± 0.108	0.677 ± 0.103	**0.004**	0.669 ± 0.102	**0.016**
FN T-Score [SD]	−1.23 ± 0.97	−1.55 ± 0.93	**0.004**	−1.62 ± 0.92	**0.016**
Osteoporotic status on hip DXA^a^					
Osteoporosis [%]	7	15		21	
Osteopenia [%]	58	63	**0.004**	52	**0.007**
Normal BMD [%]	35	22		27	
**3D DXA finite element-based strength**					
Strength Fall [N]	3563 ± 980	3234 ± 894	**0.001**	3073 ± 858	**0.001**
Strength Stance [N]	6583 ± 1574	6088 ± 1452	**0.002**	5863 ± 1404	**0.001**
**3D DXA parameters**					
TH vBMD [mg/cm^3^]	302 ± 49	286 ± 49	**0.003**	278 ± 47	**0.004**
FN vBMD [mg/cm^3^]	341 ± 67	323 ± 63	**0.006**	309 ± 61	**0.002**
TH Tb vBMD [mg/cm^3^]	162 ± 34	151 ± 34	**0.002**	145 ± 32	**0.002**
FN Tb vBMD [mg/cm^3^]	202 ± 51	188 ± 48	**0.006**	176 ± 45	**0.002**
TH Ct vBMD [mg/cm3]	830 ± 69	813 ± 70	**0.026**	812 ± 79	0.072
FN Ct vBMD [mg/cm3]	843 ± 89	820 ± 89	**0.017**	814 ± 99	**0.034**
TH Ct sBMD [mg/cm^2^]	155 ± 21	148 ± 21	**0.003**	146 ± 21	**0.016**
FN Ct sBMD [mg/cm^2^]	133 ± 22	127 ± 20	**0.014**	125 ± 21	**0.013**
TH CTh [mm]	1.859 ± 0.143	1.808 ± 0.139	**0.003**	1.791 ± 0.120	**0.004**
FN CTh [mm]	1.605 ± 0.188	1.575 ± 0.171	0.085	1.553 ± 0.176	**0.038**
FN CSA [mm^2^]	0.977 ± 0.177	0.929 ± 0.176	**0.006**	0.914 ± 0.188	**0.008**
FN CSMI [mm^4^]	1.202 ± 0.290	1.157 ± 0.317	**0.032**	1.176 ± 0.346	0.199
FN Z [mm^3^]	0.648 ± 0.142	0.616 ± 0.148	**0.004**	0.609 ± 0.162	**0.010**

DXA, dual energy x-ray absorptiometry; BMD, bone mineral density; FN, femoral neck; TH, total hip, aBMD, areal BMD; vBMD, volumetric BMD; sBMD, surface BMD; Tb, trabecular; Ct, cortical, CSA, cross sectional area; CSMI, cross-sectional moment of inertia; Z, section modulus.

Values are means ± standard deviation (SD) or number (%). P values in bold indicate results that are statistically significant (p < 0.05).

a Osteoporotic status on TH or FN aBMD, Osteoporosis defined as at least one T-score ≤ −2.5SD, and osteopenia as at least one T-score between −1 and − 2.5SD with none ≤ − 2.5SD at total hip or femoral neck.

Table 2 summarizes the HRs for the occurrence of LT clinical fractures and MOF per SD decrease in the respective parameters. All DXA and FE parameters, as well as the majority of 3D DXA parameters, were significantly associated with LT clinical fractures and MOFs. The risk of MOF was increased by 46% and 49% per one SD decrease in FN and TH aBMD, respectively. It was increased by 50% per one SD decrease in hip strength (fall) and by 57% and 59% per one SD decrease in total and Tb vBMD, respectively, at the total hip.

**Table 2:** Associations between 3D DXA-based bone strength or structural parameters of the proximal femur, and incident fractures.

	Low-trauma clinical fractures		MOF	
	HR (95%CI)	P-value	HR (95%CI)	P-value
**DXA areal BMD**				
TH aBMD	1.33 (1.09, 1.63)	**0.005**	1.49 (1.10, 2.01)	**0.010**
FN aBMD	1.37 (1.12, 1.68)	**0.002**	1.46 (1.07, 2.00)	**0.017**
**3D DXA finite element-based strength**				
Strength Fall	1.32 (1.08, 1.60)	**0.006**	1.50 (1.12, 2.01)	**0.006**
Strength Stance	1.29 (1.07, 1.57)	**0.008**	1.42 (1.08, 1.87)	**0.011**
**3D DXA parameters**				
TH vBMD	1.33 (1.09, 1.63)	**0.006**	1.57 (1.15, 2.15)	**0.004**
FN vBMD	1.23 (1.01, 1.49)	**0.038**	1.46 (1.10, 1.93)	**0.009**
TH Tb vBMD	1.31 (1.07, 1.60)	**0.008**	1.59 (1.18, 2.14)	**0.002**
FN Tb vBMD	1.24 (1.02, 1.50)	**0.031**	1.48 (1.11, 1.96)	**0.007**
TH Ct vBMD	1.22 (1.00, 1.49)	0.051	1.25 (0.92, 1.68)	0.152
FN Ct vBMD	1.22 (1.00, 1.48)	0.053	1.30 (0.96, 1.75)	0.086
TH Ct sBMD	1.34 (1.10, 1.64)	**0.004**	1.43 (1.05, 1.93)	**0.021**
FN Ct sBMD	1.24 (1.02, 1.50)	**0.032**	1.36 (1.01, 1.82)	**0.042**
TH CTh	1.38 (1.13, 1.68)	**0.002**	1.51 (1.12, 2.04)	**0.007**
FN CTh	1.10 (0.91, 1.33)	0.307	1.21 (0.91, 1.60)	0.183
FN CSA	1.26 (1.03, 1.53)	**0.023**	1.33 (0.99, 1.79)	0.055
FN CSMI	1.13 (0.94, 1.35)	0.183	1.05 (0.81, 1.37)	0.718
FN Z	1.17 (0.98, 1.40)	0.082	1.18 (0.91, 1.52)	0.205

DXA, dual energy x-ray absorptiometry; BMD, bone mineral density; FN, femoral neck; TH, total hip, aBMD, areal BMD; vBMD, volumetric BMD; sBMD, surface BMD; Tb, trabecular; Ct, cortical, CSA, cross sectional area; CSMI, cross-sectional moment of inertia; Z, section modulus; HR, hazard ratio; CI, confidence interval.

Data are hazard ratios associated with 1 SD impairment of each parameter, obtained from Cox's proportional hazard models. P values in bold indicate results that are statistically significant (p < 0.05).

The performances for fracture prediction, adjusted for age, were tested for FE-based strength, TH vBMD and Tb vBMD, in comparison with TH and FN aBMD (continuous variables, Table 3). C indices of FE-based strength, TH vBMD and Tb vBMD (0.644, 0.613, 0.624) were numerically higher than those of TH and FN aBMD (0.601, 0.599) for MOF prediction, however the differences were not statistically significant. The differences of C-indices were less pronounced for LT clinical fractures discrimination.

**Table 3:** Comparison of fracture prediction between hip aBMD and 3D DXA-based bone strength or vBMD in age-adjusted models.

	Low-trauma clinical Fractures					MOF				
	HR (95% CI)⁎	*P*-value	Harrell's C (95% CI)	P-value vs TH aBMD	P-value vs FN aBMD	HR (95% CI)⁎	*P*-value	Harrell's C (95% CI)	P-value vs TH aBMD	P-value vs FN aBMD
TH aBMD	1.34 (1.09, 1.63)	**0.005**	0.588 (0.528, 0.647)	Ref	/	1.49 (1.10, 2.01)	**0.010**	0.601 (0.510, 0.693)	Ref	/
FN aBMD	1.37 (1.12, 1.69)	**0.002**	0.591 (0.532, 0.650)	/	Ref	1.46 (1.07, 2.00)	**0.017**	0.599 (0.507, 0.691)	/	Ref
3D DXA hip strength (fall configuration)	1.32 (1.08, 1.60)	**0.006**	0.606 (0.550, 0.662)	0.390	0.404	1.50 (1.12, 2.01)	**0.006**	0.644 (0.560, 0.728)	0.254	0.104
3D DXA TH vBMD	1.33 (1.09, 1.63)	**0.006**	0.589 (0.530, 0.649)	0.897	0.918	1.58 (1.15, 2.15)	**0.004**	0.613 (0.519, 0.707)	0.608	0.642
3D DXA TH Tb vBMD	1.31 (1.07, 1.60)	**0.008**	0.597 (0.533, 0.656)	0.597	0.766	1.59 (1.18, 2.14)	**0.002**	0.624 (0.531, 0.716)	0.473	0.453

BMD, bone mineral density; FN, femoral neck; TH, total hip, aBMD, areal BMD; vBMD, volumetric BMD; Tb, trabecular; HR, hazard ratio; CI, confidence interval. P values in bold indicate results that are statistically significant (p < 0.05).

⁎ Data are hazard ratios associated with 1 SD impairment of each parameter, obtained from Cox's proportional hazard models adjusted for age.

The following analyses used categorical variables for BMD or strength thresholds. Table 4 summarizes NRIs for MOFs and LT clinical fractures for all strength thresholds ranging from 2300 N to 3000 N, when considering the aBMD thresholds (T-scores ≤ −2.5 SD) at the FN and TH. Out of values ranging from 2300 N to 2900 N, the best NRIs were found for a threshold of 2600 N (very fragile threshold). For that specific threshold, NRI for MOF was 0.114 (*p* = 0.042) and 0.220 (*p* = 0.013) for reclassification versus aBMD thresholds at the FN and TH, respectively. For the same thresholds, the NRI becomes 0.105 (*p* = 0.023) for LT clinical fractures and TH aBMD. For the fragile strength threshold (3000N), NRI for MOF were 0.020 (*p* = 0.391) and 0.039 (p = 0.391) when considering the aBMD thresholds at the FN and TH, respectively.

**Table 4:** Reclassification analyses for low-trauma clinical fracture and major osteoporotic fractures (MOF).

DXA site	3D-DXA-based FEA Threshold [N]	Low-trauma clinical fracture		MOF	
		NRI ± SE	P-value	NRI ± SE	*P*-value
**FN (T-score ≤ −2.5SD)**	2300	0.004 ± 0.010	0.720	0.042 ± 0.032	0.190
	2400	0.024 ± 0.030	0.415	0.042 ± 0.056	0.449
	2500	0.016 ± 0.030	0.583	0.086 ± 0.046	0.060
	2600	0.055 ± 0.036	0.129	0.114 ± 0.056	**0.042**
	2700	0.053 ± 0.039	0.175	0.103 ± 0.051	**0.045**
	2800	0.054 ± 0.035	0.120	0.060 ± 0.040	0.127
	2900	0.034 ± 0.020	0.099	0.021 ± 0.023	0.353
	3000	0.025 ± 0.018	0.152	0.020 ± 0.023	0.391
**TH (T-score ≤ −2.5SD)**	2300	0.020 ± 0.024	0.391	0.046 ± 0.040	0.246
	2400	0.062 ± 0.037	0.099	0.087 ± 0.061	0.150
	2500	0.066 ± 0.040	0.101	0.122 ± 0.069	0.077
	2600	0.105 ± 0.046	**0.023**	0.220 ± 0.089	**0.013**
	2700	0.095 ± 0.048	**0.047**	0.103 ± 0.069	0.136
	2800	0.083 ± 0.049	0.091	0.119 ± 0.073	0.100
	2900	0.043 ± 0.041	0.296	0.038 ± 0.046	0.410
	3000	0.028 ± 0.036	0.440	0.039 ± 0.046	0.391

Reclassification was computed using different thresholds for fragile bone strength: 3000 N as defined by Kopperdahl et al. (Kopperdahl et al., 2014), and alternative thresholds from 2300 N to 2900 N. The lower value of 2300 N was defined based on the regression between QCT- and 3D-DXA-based FEA in a recent in vitro study (Dudle et al., 2023). The need for a lower threshold with 3D-DXA images may arise from the use of solid versus liquid calibration phantoms (Faulkner et al., 1993). Reclassification between DXA and 3D-DXA-based FEA was computed when considering femoral neck (FN) or total hip (TH) based T-scores. The cutoff probabilities separating fracture risk categories were fixed at 20% for clinical fractures and 15% for MOF. NRI values that are significant at a significance level of *p* = 0.05 are printed bold.

**Fig. 1:**
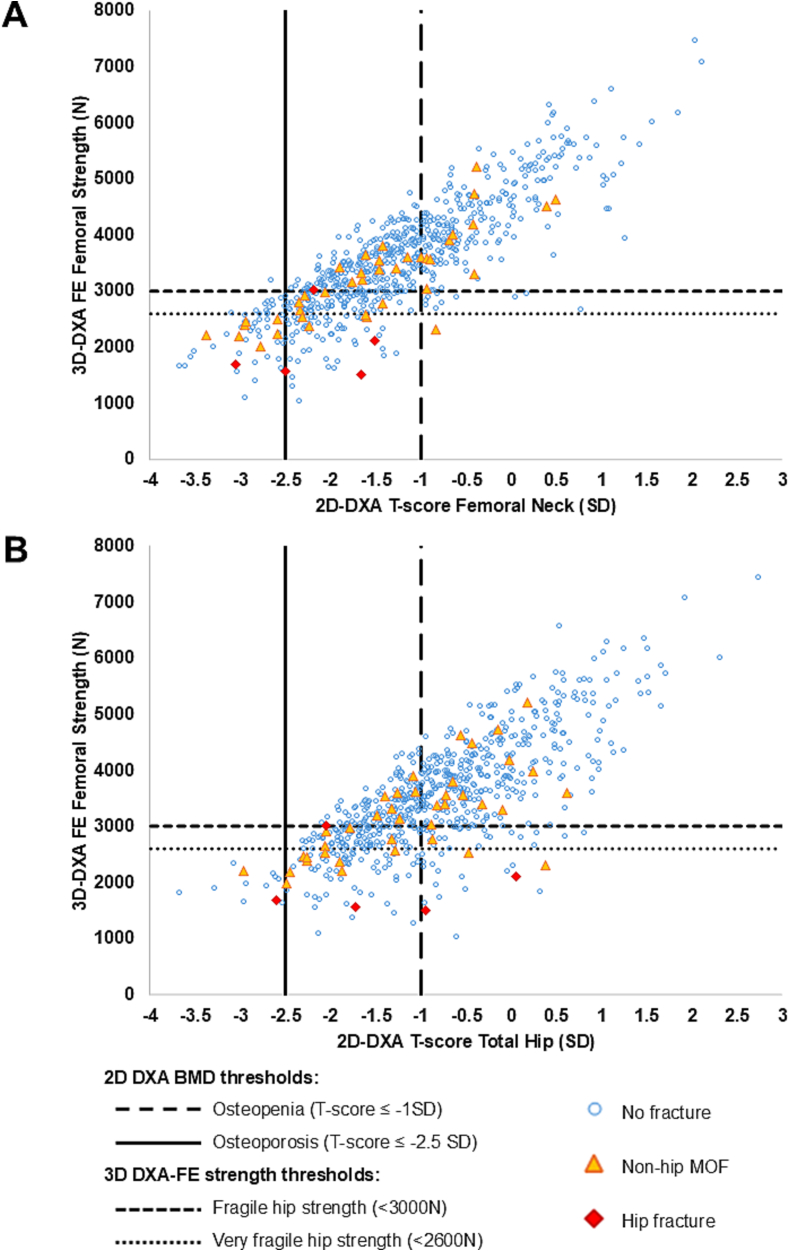
Reclassification plot of major osteoporotic fractures (MOF) showing femoral strength in a fall condition versus DXA-based T-score evaluated at the FN (A) and TH (B) with thresholds for osteoporosis (−2.5 SD) and osteopenia (−1 SD) (vertical lines), and fragile and very fragile strength (horizontal lines). Hip strength threshold: fragile <3000 N, very fragile <2600 N. Subjects who experienced a hip fracture are shown in red and a non-hip MOF in orange.

**Fig. 2:**
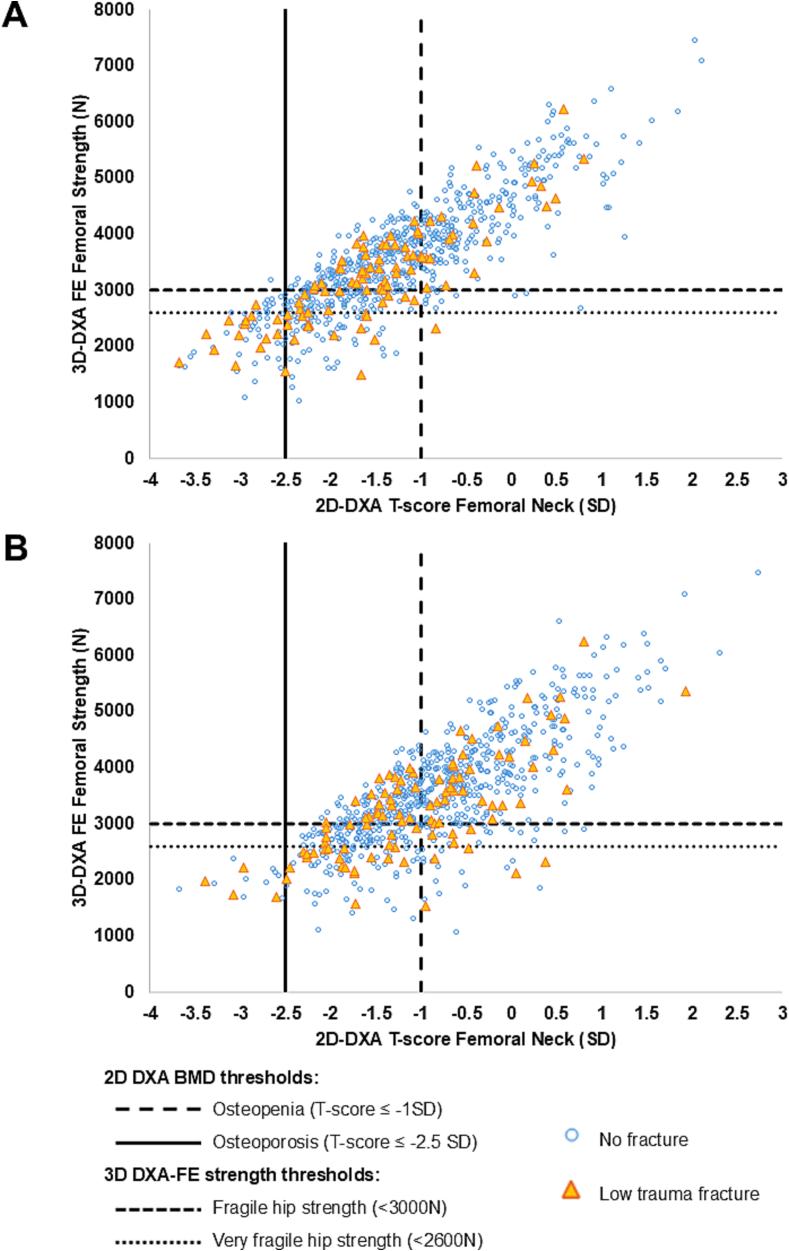
Reclassification plot of low-trauma fractures showing femoral strength in a fall condition versus DXA-based T-score evaluated at the FN (A) and TH (B) with thresholds for osteoporosis (−2.5 SD) and osteopenia (−1 SD) (vertical lines), and fragile and very fragile strength (horizontal lines). Hip strength threshold: fragile <3000 N, very fragile <2600 N. Subjects who experienced a low-trauma clinical fracture are shown in orange.

Fig. 1 shows the plot for a reclassification of individuals from FN (A) and TH (B) aBMD to femoral strength. All individuals who had osteoporotic aBMD at the FN or TH and experienced a MOF also had fragile strength according to the fragile threshold of 3000 N. Twenty-five percent of women who experienced LT fractures and 27% of women who experienced a MOF were not osteoporotic (T-score > −2.5SD at the FN or TH) but had fragile strength (≤3000 N). The respective proportions were 14% and 16% for very fragile strength (≤2600 N). Notably, finite element analysis revealed that all five hip fracture cases exhibited femoral strength values within the fragile range or at its threshold, regardless of their aBMD status (osteoporotic, osteopenic or normal at FN or TH). Four out of five hip fracture cases were captured as very fragile (≤2600 N). Five subjects with MOFs among the 20 ones with normal BMD (T-score > -1DS, 25%) at the TH were also reclassified with fragile strength. Reclassification plots for all LT fractures are shown in Fig. 2.

Table 5 summarizes the performances for fracture prediction of various thresholds of strength and aBMD. FN aBMD (T-scores ≤ −2.5SD) was the best predictor of MOFs, with a C-index of 0.629. Very fragile strength, as defined by the threshold of 2600 N (C-index 0.570) did not perform better than FN aBMD threshold (*p* = 0.458), but it did compared to TH aBMD threshold (C-index 0.507, *p* = 0.039). The picture is similar for the prediction of LT clinical fracture (C-indices: strength 0.603 vs FN aBMD 0.580, *p* = 0.304; vs TH aBMD 0.538, *p* = 0.025).

**Table 5:** Comparison of fracture prediction between hip aBMD and 3D DXA-based bone strength, using binary variables according to different thresholds of aBMD and strength, in age-adjusted models.

Variables included in the models⁎	Low-trauma clinical Fractures						MOF					
	HR (95% CI)⁎	*P*-value	Harrell's C (95% CI)⁎	P-value vs TH T-score < −2.5DS	P-value vs FN T-score < −2.5DS	P-value vs TH or FN T-score < −2.5DS	HR (95% CI)⁎	P-value	Harrell's C (95% CI)⁎	P-value vs TH T-score < −2.5DS	P-value vs FN T-score < −2.5DS	P-value vs TH or FN T-score < −2.5DS
TH aBMD T-score < −2.5DS	2.25 (0.82, 6.15)	0.115	0.538 (0.478, 0.599)	Ref	/	/	2.63 (0.63, 10.99)	0.184	0.506 (0.411, 0.602)	Ref	/	/
FN aBMD T-score < −2.5DS	2.20 (1.27, 3.81)	**0.005**	0.580 (0.519, 0.640)	/	Ref	/	3.12 (1.49, 6.50)	**0.002**	0.629 (0.538, 0.719)	/	Ref	/
TH or FN aBMD T-score < −2.5DS	2.04 (1.18, 3.54)	**0.011**	0.579 (0.519, 0.640)	/	/	Ref	2.90 (1.39, 6.04)	**0.004**	0.525 (0.419, 0.631)	/	/	Ref
Strength <3000 N	1.40 (0.94, 2.09)	0.101	0.576 (0.521, 0.631)	0.234	0.898	0.906	1.85 (1.02, 3.36)	**0.044**	0.566 (0.457, 0.676)	0.107	0.417	0.252
Strength <2600 N	2.07 (1.33, 3.21)	**0.001**	0.603 (0.545, 0.662)	**0.025**	0.304	0.298	2.93 (1.57, 5.46)	**0.001**	0.570 (0.457, 0.682)	**0.039**	0.458	0.111

P values in bold indicate results that are statistically significant (p < 0.05).

⁎ Adjusted for age.

**Fig. 3:**
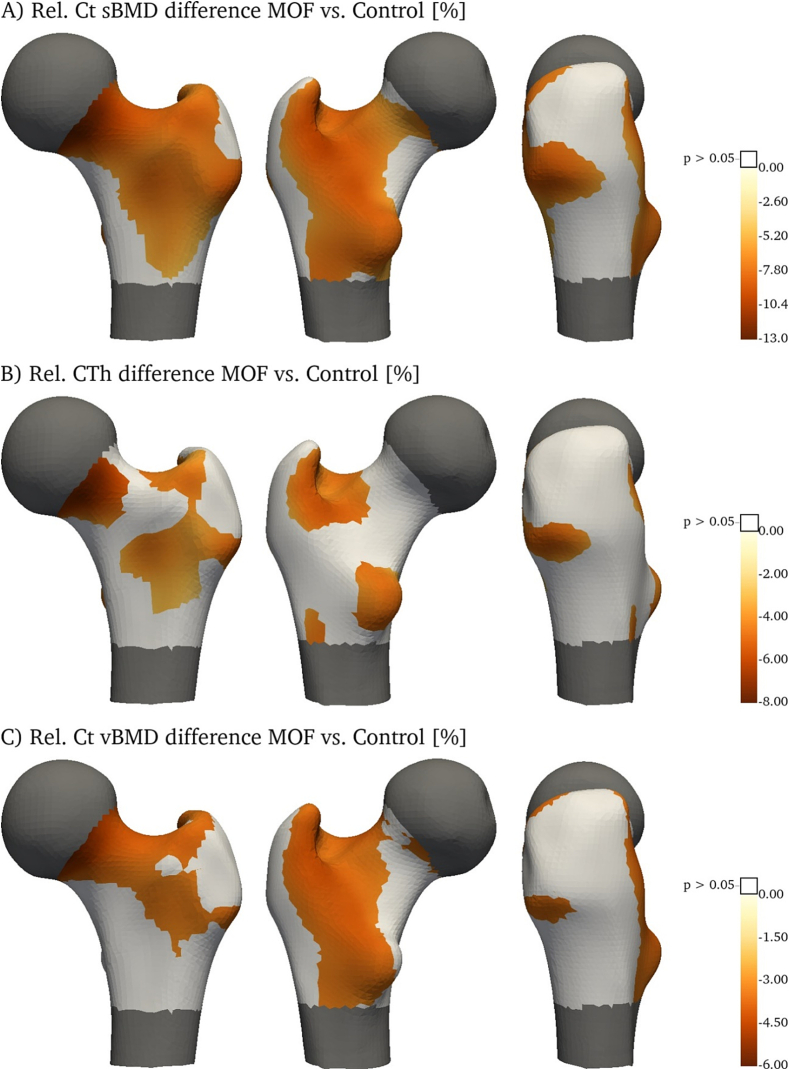
Relative differences (%) in cortical parameters – sBMD (A), CTh (B) and Ct vBMD (C) – between women who will sustain a major osteoporotic fracture (MOF) and women without incident fracture (control). Areas without significant differences are printed white.

**Fig. 4:**
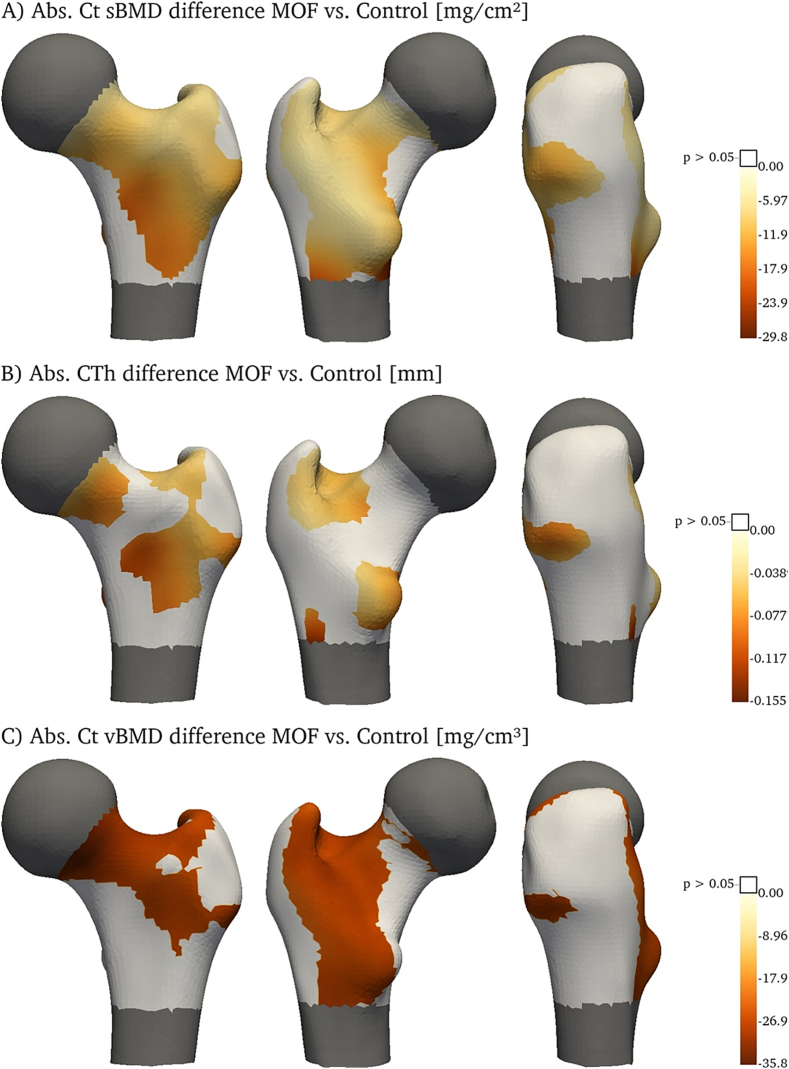
Absolute difference in mean cortical parameters – sBMD (A), CTh (B) and Ct vBMD (C) – between women who will sustain a major osteoporotic fracture (MOF) and women without incident fracture (control). Areas without significant differences are printed white.

Fig. 3 illustrates the distribution of the relative differences in Ct sBMD (A), CTh (B) and Ct vBMD (C) between women who sustained a MOF and those without fracture (controls). Areas with non-significant differences (*p* > 0.05) are printed white. Women with MOFs had significantly lower CTh in the anterior part of the FN, in the trochanteric fossa and around the lesser trochanter. Another area with markedly decreased CTh is found around the intertrochanteric line and extends laterally to the GT. Subjects experiencing MOF had significantly lower Ct vBMD from the antero-superior FN, extending through the trochanteric fossa and along the intertrochanteric line down to the proximal lateral side of the lesser trochanter and extending distally. The areas with significant differences in Ct sBMD seem to be close to the combination of the areas showing significant differences in CTh or Ct vBMD. This may reflect the close connection between these parameters. Absolute differences are shown in Fig. 4.

## 4 Discussion

To our knowledge, our study provides the first prospective analysis of any fracture prediction with hip bone strength assessed using 3D DXA-based FEA with 3D-SHAPER software, in postmenopausal women. In this population-based cohort of postmenopausal women, 3D DXA-based FEA and most structural parameters of the hip predicted incident MOFs and LT clinical fractures. Small improvements were even observed through a reclassification analysis when applying a strength-based threshold of 2600 N when compared to FN or TH aBMD-based thresholds at T-scores lower than −2.5SD. There was no improvement in fracture prediction in models using femoral strength and BMD as continuous variables. It is noteworthy that approximately 25% of women who experienced a fracture had no hip osteoporosis (T-score ≤ −2.5), yet exhibited compromised mechanical resistance, characterized by fragile (〈3000N) or very fragile (<2600 N) hip strength. In addition, a comparison of the distribution of local cortical parameters between subjects without incident fractures and subjects who sustained a MOF (non-hip for the majority) showed significant differences between the groups, notably at the femoral neck and intertrochanteric regions. These observations strengthen the notion that women with a history of non-hip fractures represent a subgroup at elevated risk for sustaining subsequent hip fractures.

Several cross-sectional studies have shown that structural cortical and trabecular parameters measured from DXA-derived 3D models discriminated between fracture and control groups (Ruiz Wills et al., 2019; Humbert et al., 2020). However, few data are available regarding the prediction of incident fractures using 3D DXA models. Associations between LT MOFs and bone strength or vBMD, obtained from opportunistically screened CT images, have been published before. These studies indicated that hip strength assessed with QCT-based FE analyses worked equally well as hip aBMD to predict prevalent fractures (Amin et al., 2011; Michalski et al., 2021). A recent study showed that 3D-DXA-FE modeling using 3D-Shaper software provided accurate femur strength estimates compared to QCT-FE models (R^2^ = 0.86) (Qasim et al., 2024).

Therefore, 3D DXA-based FEA may provide accurate femur strength estimates without requiring additional tests or radiation exposure. The finite element (FE) simulation of a proximal femur takes approximately one hundred seconds on about 8–16 central processing units of the computing cluster, indicating that the time required to complete a FEA with each 3D scan would not be prohibitive for the routine use of this technology.

Fracture cases in our study (3D-DXA-based) and QCT-based studies reported above were dominated by fractures of the upper limb (radius, humerus). A prospective analysis in the Osteoporotic Fractures in Men study showed that estimated femoral strength using another FE model from DXA scans of the proximal femur provided limited improvement in the predictive ability for FN fracture only, despite focusing on hip fracture, occurring at the same bone site of bone parameters assessment (Yang et al., 2018). Grassi et al. reported an improved AUC for the prediction of hip fractures based on FE models built from reconstructed 3D images, the improvement was specifically pronounced in a sub-group of fallers (Grassi et al., 2023; Grassi et al., 2025). For the 2D-to-3D reconstruction, the authors utilized mesh-based SSAM, a method distinct from the approach implemented in the 3D-Shaper software.

Another prospective study conducted in a Japanese population-based osteoporosis cohort using the 3D-SHAPER software reported that vBMD derived from 3D hip DXA modeling significantly improved hip fracture risk prediction compared with conventional FN aBMD, but not TH aBMD, opposite to what we observed (Iki et al., 2021). Consistent with our findings, Tb vBMD was among the parameters most strongly associated with fracture risk. The discrepancy between FN and TH observed across this study and ours may reflect the greater contribution of Tb bone to non-hip fractures, which is likely better captured at the TH site. The number of hip fractures (*n* = 5) in our study was too small to confirm such a finding. Nonetheless, hip fractures were without exception close or below a previously defined threshold for fragile bone strength (3000 N), regardless their FN or TH aBMD values, which ranged from osteoporotic to normal (Fig. 1) (Kopperdahl et al., 2014).

While the use of strength-based thresholds improved the identification of fracture cases compared to aBMD criteria (C-indices in Table 5), their application would also result in upward risk reclassification among numerous individuals without fractures (Fig. 1, Fig. 2). This overclassification may limit the specificity and overall discriminative performance of hip strength thresholds in LT clinical fracture risk assessment.

The best reclassification results were obtained with a threshold for fragile bone strength of 2600 N, whereas a former study established a threshold of 3000 N for fragile femoral strength in women (Kopperdahl et al., 2014). The statistical model of 3D-Shaper was built based on a dataset of CT images that were calibrated with a K_2_HPO_4_ equivalent calibration phantom, whereas a solid hydroxyapatite calibration phantom had been used in the CT dataset that was used for establishing the threshold for fragile bone strength (Humbert et al., 2017; Kopperdahl et al., 2014). Parts of the differences may be due to differences in the FE modeling approach or could be due to a cohort effect. Nonetheless, density calibration may play an important role. In a cross-calibration study, Faulkner et al. reported lower density values for liquid than for solid phantoms (Faulkner et al., 1993). If 3D DXA images will be used alongside CT images for FEA and fracture risk prediction in the future, care should be taken with respect to these calibration procedures.

Maps of cortical surface properties showed areas with significant differences between controls and subjects who sustained a MOF. Similar analyses were performed based on CT images for the comparison of hip fracture cases with control subjects (Poole et al., 2017). This study also found the largest Ct sBMD difference in the supero-anterior part of the FN and found extended areas with significant thickness differences in the anterior part of the proximal femur. However, no differences were found along the intertrochanteric line and around the lesser trochanter.

We acknowledge several limitations to our findings. An important limitation of the current study is that we did not exploit all the information available from the 3D DXA images to build the FE models. In fact, voxel-based FE models do not allow to correctly capture the structural morphology of the femur with a thin cortical shell and the trabecular zone. 3D-Shaper offers information on cortical and trabecular compartments. By building models that incorporate this information, the mechanical behavior of the bone may be captured more precisely. However, this improvement is likely to come at an increased computational cost. In addition, while this work applied FEA in two loading scenarios, many other loading scenarios exist which may yield different prediction of fractures. A total of 64 densitometric and structural parameters of the proximal femur are evaluated by the 3D-Shaper software. The analyses considered the 13 main parameters at the total hip or femoral neck. The population studied here represented a younger population which may not represent the highest risk individuals, with a low number of hip fractures, so that it did not allow to assess the predictive power of 3D DXA and FEA for hip fracture prediction alone, which may be the main field of application for 3D-Shaper.The findings of this exploratory study need therefore to be replicated in more heterogeneous and older populations at higher risk of hip fracture. The limited follow-up time is another limiting factor from this perspective. A longer follow-up time, e.g., 10 years, could add power and may allow to better evaluate whether low estimated strength is a significant factor beyond DXA.

In conclusion, hip strength assessed with 3D DXA-based FEA and structural hip parameters obtained with 3D DXA predict incident LT fractures and MOF in postmenopausal women. These data indicate that the well-established association between hip DXA parameters and fracture risk is not altered by the software algorithm used by DXA-based 3D modeling. A femoral strength threshold of 2600 N, derived from 3D DXA-based finite element analysis, demonstrated superior fracture predictive performance compared to conventional 2D DXA aBMD thresholds at the femoral neck and total hip (T-score ≤ −2.5 SD). Marked regional variations in cortical bone characteristics were observed between women presenting with incident predominantly non-hip MOF and control subjects. These results suggest that biomechanical evaluation of the proximal femur can provide complementary information to aBMD and improve identification of individuals at elevated fracture risk. Incorporating strength-based assessment into routine evaluation may enhance preventive strategies, particularly for patients who do not meet osteoporosis diagnostic thresholds.

## CRediT authorship contribution statement

**Yvan Gugler:** Conceptualization, Data curation, Formal analysis, Investigation, Methodology, Writing – original draft. **Philippe Zysset:** Conceptualization, Funding acquisition, Project administration, Resources, Supervision, Validation, Visualization, Writing – review & editing. **Serge Ferrari:** Conceptualization, Funding acquisition, Project administration, Resources, Validation, Visualization, Writing – review & editing. **Emmanuel Biver:** Conceptualization, Data curation, Formal analysis, Funding acquisition, Investigation, Project administration, Supervision, Validation, Visualization, Writing – review & editing.

## Funding

This project was made possible through a SINERGIA Grant (grant number 183584) from the 10.13039/501100001711Swiss National Science Foundation (Schweizerischer Nationalfonds zur Förderung der Wissenschaftlichen Forschung). The GERICO cohort was supported by grants from the 10.13039/501100006388Geneva University Hospitals and Faculty of Medicine Clinical Research Center, the HUG Private Foundation, the BNP-Paribas Foundation, and the Fondation pour la Recherche sur l'Ostéoporose et les Maladies Osseuses de Genève.

## Declaration of competing interest

The authors declare the following financial interests/personal relationships which may be considered as potential competing interests: Philippe Zysset and Serge Ferrari reports financial support was provided by Swiss National Science Foundation. Serge Ferrari and Emmanuel Biver reports financial support was provided by Geneva University Hospitals and Faculty of Medicine Clinical Research Center. Serge Ferrari and Emmanuel Biver reports financial support was provided by HUG Private Foundation. Serge Ferrari and Emmanuel Biver reports financial support was provided by FROMO Genève. If there are other authors, they declare that they have no known competing financial interests or personal relationships that could have appeared to influence the work reported in this paper.

## Data Availability

The authors do not have permission to share data.

## Appendix A Supplementary data

Supplementary material Image 1
