# Supplementary material for: Fracture prediction using 3D-DXA-finite element based femoral strength: a prospective study in postmenopausal women
Source: Bone Rep. 2026 Feb 10;29:101906. doi: 10.1016/j.bonr.2026.101906 (PMC12925575; doi:10.1016/j.bonr.2026.101906)
Supplement: Supplementary file 1 — Supplementary material [file mmc1.pdf]

Supplementary Materials

<u>Supplementary Figure 1</u>: A) Regions for 3D DXA parameters: femoral neck (orange), trochanter (turquoise), shaft (blue). B-C) Example Finite Element Meshes in fall (B) and stance configuration (C)- For the stance load case the proximal shaft axis was inclined by 20° with respect to the vertical axis.

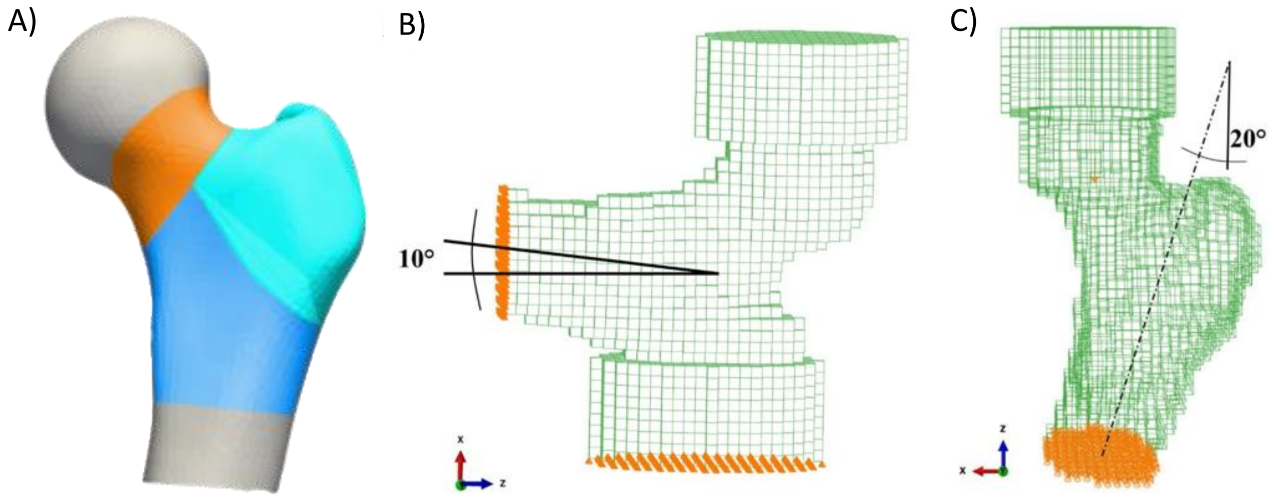

Supplementary Figure 2: Study flowchart

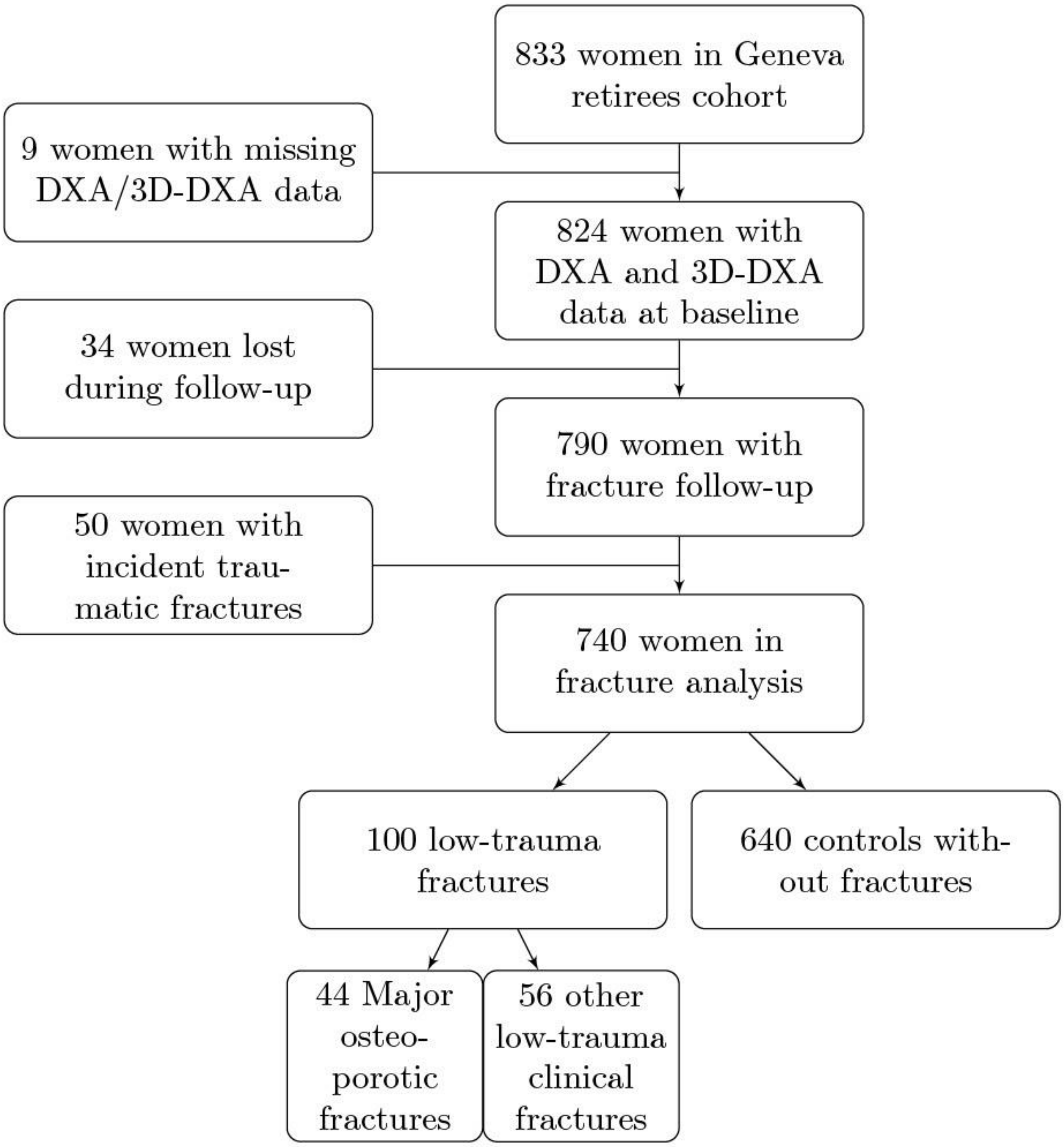

Supplementary Table 1: List of parameters analyzed in the study

Parameter	Abbreviation	Technique	Unit
Total hip areal BMD	TH aBMD	DXA	[g/cm^2]
Femoral neck areal BMD	FN aBMD		
Trochanteric areal BMD	Troch aBMD		
Shaft areal BMD	Shaft aBMD		
Total hip volumetric BMD	TH vBMD	3D-DXA	[mg/cm^3]
Femoral neck volumetric BMD	FN vBMD		
Total hip trabecular volumetric BMD	TH Tb vBMD		
Femoral neck trabecular volumetric BMD	FN Tb vBMD		
Total hip cortical volumetric BMD	TH Ct vBMD		
Femoral neck cortical volumetric BMD	FN Ct vBMD		
Total hip surface BMD	TH sBMD		[mg/cm^2]
Femoral neck surface BMD	FN sBMD		
Total hip cortical thickness	TH CTh		[mm]
Femoral neck cortical thickness	FN CTh		
Femoral neck bone-weghted cross-sectional area	FN CSA		
Femoral neck bone-weighted cross-sectional moment of inertia	FN CSMI		[mm^4]
Femoral neck section modulus	FN Z		[mm^3]
Strength in fall configuration	Strength fall	3D-DXA FEA	[N]
Strength in stance configuration	Strength stance		

<u>Supplementary Table 2</u>: Fracture cases in the GERICO cohort. 44 subjects experienced a major osteoporotic fracture. 56 subjects experienced another low-trauma clinical fracture. 3 subjects experienced multiple fractures

Fracture classification	Bone site
**Major osteoporotic fractures (n=44)**	- Forearm (n=19)<br>- Proximal humerus (n=16)<br>- Proximal femur (n=5)<br>- Vertebrae (n=4)
**Other low-trauma clinical fractures (n=59)**	- Ankle (n=14)<br>- Metatarsal bone (n=12)<br>- Rib (n=7)<br>- Carpal bone (n=6)<br>- Tarsal/calcaneum (n=4)<br>- Lower limb (Tibia/Fibula) (n=4)<br>- Elbow (n=3)<br>- Patella (n=2)<br>- Metacarpal bone (n=2)<br>- Femur diaphysis (n=2)<br>- Pelvis (n=2)<br>- Collar bone (n=1)
